# Branched-chain polyamines: evolutionary adaptation and biotechnological potential

**DOI:** 10.1007/s00726-026-03506-4

**Published:** 2026-03-05

**Authors:** Shinsuke Fujiwara, Wakao Fukuda

**Affiliations:** 1 Department of Biosciences, School of Biological and Environmental Sciences, Kwansei-Gakuin University, 1 Gakuen-Uegahara, Sanda, Hyogo 669-1330 Japan; 2 NITE Biological Resource Center (NBRC), National Institute of Technology and Evaluation, 2-5-8 Kazusakamatari, Kisarazu, Chiba 292-0818 Japan

**Keywords:** Agmatine, Archaea, Branched-chain polyamine, Polyamine, *Thermococcus kodakarensis*, Thermophiles, Thermospermine, *Thermus thermophilus*

## Abstract

Branched-chain polyamines (BCPAs), exemplified by *N*⁴-bis(aminopropyl)spermidine, are distinctive polycations that occur predominantly in thermophilic bacteria and euryarchaeal archaea. Their dedicated aminopropyltransferase, BpsA (EC 2.5.1.128), extends spermidine into branched architectures via sequential decarboxylated *S*-adenosylmethionine (dcSAM)-dependent reactions. Accumulated evidence demonstrates that BCPAs engage nucleic acids with substantially higher affinity than linear polyamines such as spermidine, and they uniquely induce strong DNA compaction accompanied by B→A→C structural transitions. These interactions greatly enhance the resistance of DNA to thermal, chemical, and physical damage. Genetic and physiological analyses in *Thermococcus kodakarensis* further show that loss of BCPA biosynthesis compromises growth at very high temperatures, disrupts temperature- and membrane-associated stress responses, and alters transcriptional and translational regulation; intriguingly, the linear tetraamine thermospermine can partially substitute for BCPA in several of these functions. Beyond cellular physiology, immobilized BCPAs enable sensitive nucleic-acid capture and direct PCR and isothermal DNA amplification from highly dilute solutions, demonstrating their potential utility in molecular diagnostics and environmental DNA workflows. This review synthesizes current knowledge of BCPA distribution, biosynthesis, structure–function relationships, cellular roles, and emerging biotechnological applications, and highlights key open questions in the field.

## Discovery and distribution of branched-chain polyamines

**Fig. 1:**
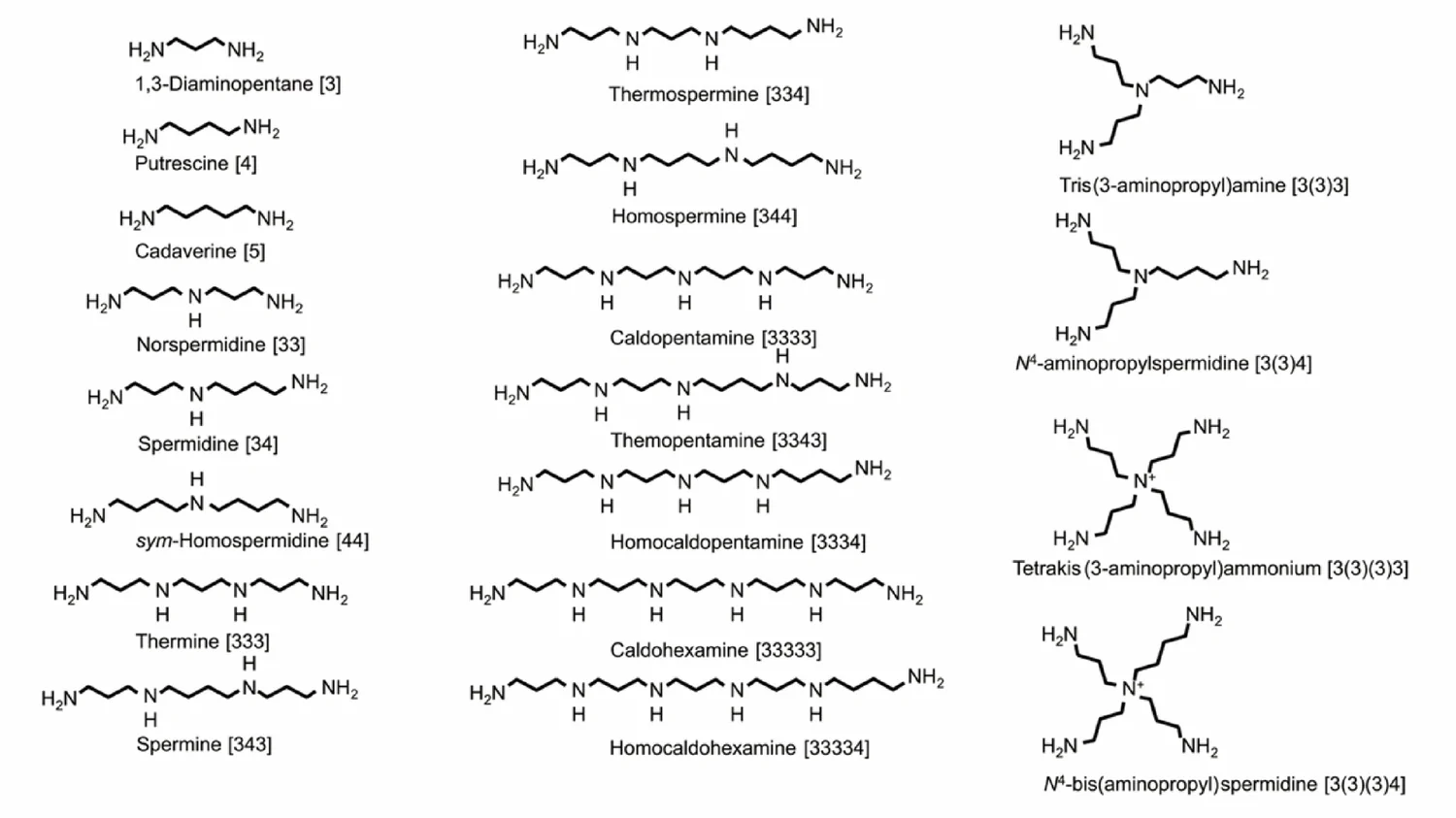
Polyamines identified in microorganisms. Chemical structures of linear and branched polyamines are shown. Abbreviation for the number of methylene CH_2_ chain units between NH_2_, NH, and N^+^

Polyamines are ubiquitous small cationic molecules that support a wide range of biological functions across all domains of life (Tabor and Tabor 1985; Wallace et al. 2003; Michael 2016). Polyamines are abundant in rapidly proliferating cells, including cancers, where they stabilize nucleic acids and promote gene expression (Casero and Pegg 2009; Pegg 2016). Early work by Hamana, Niitsu, and colleagues in the 1980–1990 s played a pioneering role in expanding the known diversity of polyamines. Using the best analytical technologies available at the time, they reported that, in addition to canonical linear polyamines, many thermophilic and hyperthermophilic microorganisms synthesize unusual long-chain polyamines (LCPAs) and branched-chain polyamines (BCPAs) (Hamana et al. 1991a, 1993, 1994, 1996a, 1999, 2007, 2022) as shown in Fig. 1. Their surveys also extended to insects, algae, and plants, greatly broadening the conceptual landscape of polyamine chemistry. BCPAs, however, consistently appeared in thermophiles and hyperthermophiles, suggesting an ecological and evolutionary association with high-temperature environments. Within this context, earlier reports described the detection of branched penta-amines—such as *N*⁴-bis(aminopropyl)spermidine [3(3)(3)4] and *N*⁵-aminobutylhomospermine [4(4)44] (where the number in square parentheses represents the number of methylene ­ CH_2_ chain units between ­ NH_2_, NH, N, or ­ N^+^) in concentrated acid-extractable fractions from legume seeds (Hamana et al. 1991b, 1992, 1996b). These findings were entirely reasonable given the analytical sensitivity and column technologies of that period, which made trace-level differentiation challenging and background signals difficult to eliminate completely. Subsequent analyses using more advanced chromatographic and MS methods, however, did not reproduce these detections in freshly collected seeds (Otsuka et al. 2005). A recent re-evaluation by Hamana and Niitsu themselves (2025) concluded that earlier observations were likely influenced by minor sample preparation artifacts, such as trace carry-over during concentration or column reuse, and therefore the natural occurrence of these branched penta-amines in plant seeds remains unconfirmed. Rather than diminishing the significance of the early studies, these findings underscore both the technical challenges faced at the time and the pioneering efforts of Hamana and colleagues, whose work laid the foundation for subsequent advances in polyamine analytical chemistry and understanding of polyamine diversity.

Initially, LCPAs were proposed as essential factors for survival at elevated temperatures because they were detected in Desulfurococcales and certain *Pyrobaculum* species. However, later biochemical and genomic studies revealed that LCPAs are rare or absent in many hyperthermophilic lineages; instead, BCPAs and the linear tetraamine norspermine dominate the polyamine pools of numerous hyperthermophiles (Fukuda et al. 2022). In *Thermus thermophilus*, both LCPAs and BCPAs coexist (Oshima 2010; Nakashima et al. 2017), raising intriguing questions about the physiological rationale behind maintaining a diverse polyamine composition in thermophiles. Overall, the early discovery phase established BCPAs as unique molecular signatures of organisms thriving in extreme thermal environments. This foundation paved the way for subsequent mechanistic and functional studies that have elucidated the biosynthetic logic, biochemical properties, and physiological significance of these thermophile-specific polyamines.

## Biosynthetic pathways of polyamines in prokaryotes

### Classical biosynthetic pathways of linear polyamines

**Fig. 2:**
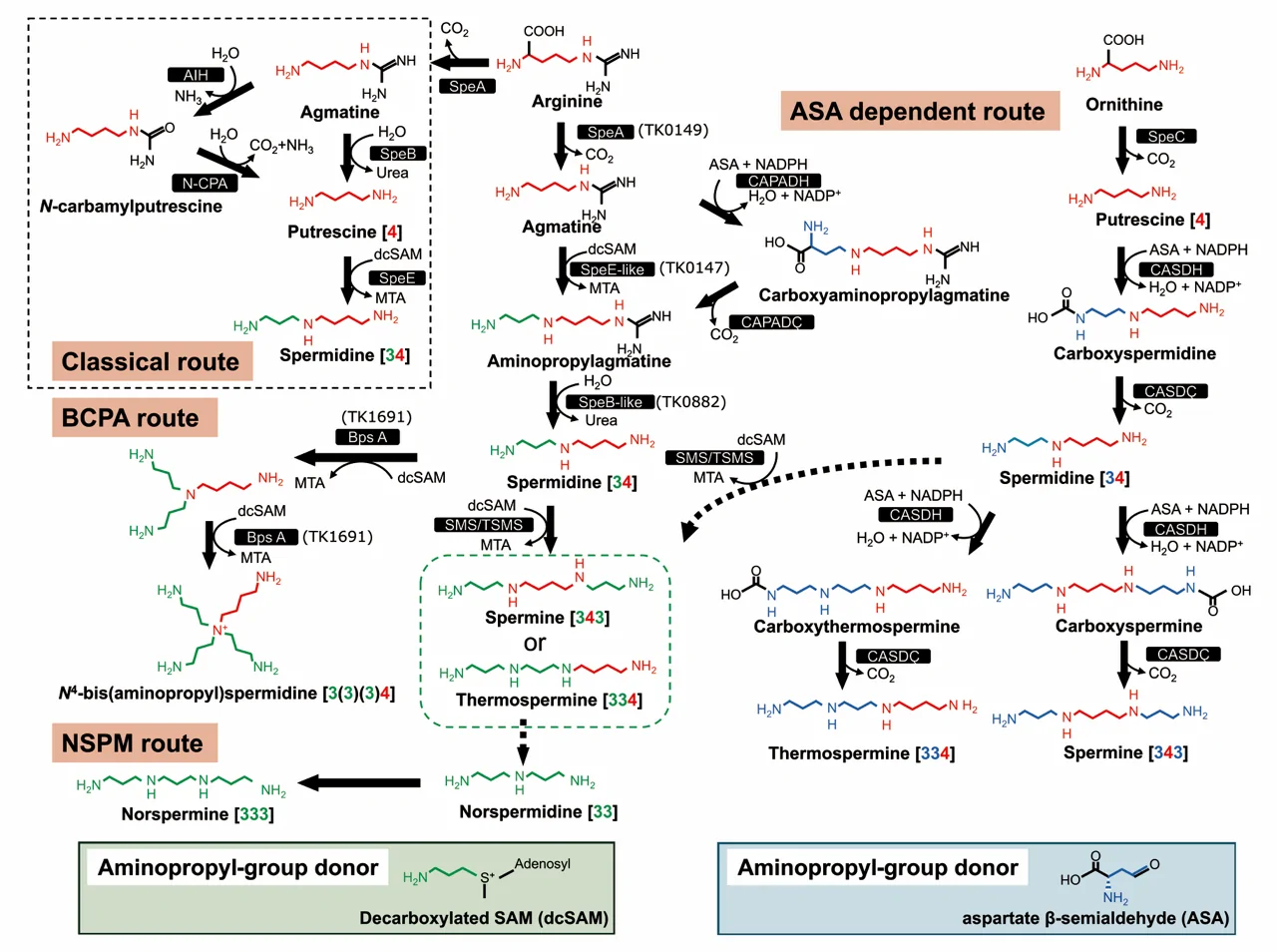
Biosynthetic pathways of polyamines, including BCPAs, in microorganisms. Polyamine biosynthetic pathways (classical, BCPA, NSPM and ASA-dependent routes) in microorganisms are shown. The classical pathway is outlined with a black dotted line. The route for BCPA synthesis is indicated as the “BCPA route.” The ASA-dependent pathway, originally identified in *Agrobacterium tumefaciens*, is shown in the right panel. Spermine and thermospermine, which are produced in *Thermus thermophilus*, are highlighted by a green dotted line. The pathway for the conversion of thermospermine to norspermine identified in *Pyrobaculum calidifontis* is also shown. In NSPM route Norspermidine was produced from thermospermine by an unidentified polyamine oxidase/dehydrogenase (broken arrow). Aminopropyl groups derived from dcSAM and ASA are indicated in green and blue, respectively. Aminobutyl groups are indicated in red. Decarboxylated S-adenosylmethionine (dcSAM) is produced from -adenosylmethionine (AdoMet) by S-adenosylmethionine decarboxylase (AdoMetDC). Broken arrows indicate pathways catalyzed by unidentified enzymes. *AIH* agmatine iminohydrolase, *ASA* aspartate β-semialdehyde, *BCPA* branched-chain polyamine, *BpsA* branched-chain polyamine synthase (aminopropyltransferase), *CAPADC* carboxyaminopropylagmatine decarboxylase, *CAPADH* carboxyaminopropylagmatine dehydrogenase, *CASDC* carboxyspermidine decarboxylase, *CASDH* carboxyspermidine dehydrogenase, *dcSAM* decarboxylated -adenosylmethionine, *N-CPA* N-carbamoylputrescine amidohydrolase, *SpeA* arginine decarboxylase, SpeB agmatinase (agmatine ureohydrolase), SpeB-like agmatine ureohydrolase–like enzyme (TK0882), *SpeC* ornithine decarboxylase, *SpeE* spermidine synthase (aminopropyltransferase), *SpeE-like* aminopropylagmatine synthase (aminopropyltransferase) (TK0147), *SPM* spermine synthase (aminopropyltransferase), *TSMS* thermospermine synthase (aminopropyltransferase). Numbers in parentheses indicate gene numbers in *Thermococcus kodakarensis*

In the history of polyamine research, the best-characterized and most widely distributed pathway is the linear polyamine biosynthetic route that converts ornithine and arginine into putrescine, and subsequently into spermidine and spermine (Michael 2016, 2017; Li et al. 2025). In many bacteria and eukaryotes, ornithine decarboxylase (ODC; typically encoded by *speC* or *speF*) catalyzes the direct formation of putrescine from ornithine. In addition, in some organisms, arginine decarboxylase (ADC; often encoded by *speA*) converts arginine into agmatine, which is subsequently processed by agmatine ureohydrolase (SpeB/AguA; EC 3.5.3.11) to yield putrescine. Besides this pathway, agmatine iminohydrolase (also referred to as agmatine amidinohydrolase; AIH; EC 3.5.3.12) converts agmatine into *N*-carbamoylputrescine, which is further converted to putrescine by *N*-carbamoylputrescine amidohydrolase (N-CPA; EC 3.5.1.53) (Nakada et al. 2001), as shown in Fig. 2. Thus, AIH requires the partner enzyme N-CPA to complete the conversion of agmatine to putrescine. In bacteria, this AIH-dependent pathway contrasts with the more widely distributed agmatine ureohydrolase (SpeB) route and is found in a limited and patchy subset of lineages, most notably among Rhizobiales and several other α-proteobacteria, with additional reports in γ-Proteobacteria such as *Pseudomonas aeruginosa* and selected members of the Bacillota (Nakada et al. 2001; Griswold et al. 2009).　Notably, this pathway is not restricted to bacteria. In plants, an arginine decarboxylase-AIH-N-CPA pathway operates as an alternative route for putrescine biosynthesis and is thought to have been acquired via endosymbiotic gene transfer from the cyanobacterial ancestor of the chloroplast (Piotrowski et al. 2003; Fuell et al. 2010).Together, these observations indicate that while the AIH pathway shows a patchy and lineage-specific distribution in bacteria, it has been stably integrated and functionally retained in the plant lineage.

The conversion of putrescine into spermidine and then into spermine proceeds through a series of aminopropyl-transfer reactions that use decarboxylated *S*-adenosylmethionine (dcSAM), generated from *S*-adenosylmethionine (AdoMet), as the aminopropyl donor. *S*-adenosylmethionine decarboxylase (AdoMetDC; *speD*) first produces dcSAM. Spermidine synthase (SpeE; *speE*) then transfers the aminopropyl moiety from dcSAM to putrescine, forming spermidine. Subsequently, spermine synthase (often annotated as spermine synthase or *speG* depending on the organism) catalyzes the addition of another aminopropyl group to spermidine to generate spermine. This dcSAM-dependent aminopropyl-transfer mechanism is highly conserved across domains of life and constitutes the core “classical” polyamine biosynthetic pathway (Fig. 2).

### A thermophile-specific pathway: agmatine → *N*¹-aminopropylagmatine → spermidine

In contrast to the classical route, some thermophilic bacteria and hyperthermophilic archaea employ a distinct agmatine-based pathway for spermidine biosynthesis. In this pathway, arginine is first converted to agmatine by a pyruvoyl-dependent arginine decarboxylase, a step shared with the classical route. However, the downstream reaction order differs markedly. Instead of proceeding via deimination to putrescine, agmatine first undergoes aminopropylation, producing *N*¹-aminopropylagmatine, which is then deiminated to produce spermidine. Consequently, putrescine is barely detectable or entirely absent in these organisms. In the thermophilic bacterium *Thermus thermophilus*, the details of this pathway were first elucidated using combined genetic and biochemical approaches (Ohnuma et al. 2005; Oshima 2010). Later, the same pathway was confirmed in the hyperthermophilic archaeon *Thermococcus kodakarensis* (Morimoto et al. 2010). In *T. kodakarensis*, PdaD (TK0149/pdaD/speA/adc) is the essential enzyme that catalyzes the formation of agmatine from arginine; a *pdaD* deletion mutant cannot grow without exogenous agmatine (Fukuda et al. 2008). Importantly, agmatine is not required solely as a precursor for spermidine biosynthesis. Archaeal tRNAs utilize agmatine to generate agmatidine, an agmatine-conjugated cytidine modification that is indispensable for accurate decoding of the AUA isoleucine codon. Thus, the absence of agmatine disrupts agmatidine formation and compromises translational fidelity (Ikeuchi et al. 2010). The aminopropyltransferase TK0147 (*speE*) was originally annotated as a classical spermidine synthase, but kinetic analysis revealed that it exhibits much higher catalytic efficiency toward agmatine than putrescine, producing *N*¹-aminopropylagmatine. Furthermore, the agmatine-ureohydrolase–like enzyme TK0882 (*speB*-like agmatinase) strongly prefers *N*¹-aminopropylagmatine over agmatine and hydrolyzes it to spermidine (Morimoto et al. 2010). Thus, TK0882 (*speB*-like) is the major enzyme responsible for the final step of this pathway. In summary, the hyperthermophilic pathway in *T. kodakarensis* proceeds as: Arg → Agm (PdaD; TK0149/*speA*) → *N*¹-aminopropylagmatine (TK0147/*speE*) → Spd (TK0882/*speB*-like). This order—aminopropylation before deimination—is reversed relative to the classical pathway. This modified architecture is a remarkable thermophile-specific evolutionary innovation (Fig. 2).

### Alternative pathways via carboxyspermidine and carboxyspermine

The diversity of polyamine biosynthesis is further illustrated by pathways in which carboxylated intermediates serve as precursors for spermidine and spermine. In organisms lacking SpeE, spermidine can be synthesized through a two-step route involving carboxyspermidine dehydrogenase (CASDH) and carboxyspermidine decarboxylase (CASDC). CASDH forms carboxyspermidine from putrescine and aspartate β-semialdehyde (ASA), and CASDC subsequently decarboxylates carboxyspermidine to produce spermidine—without the use of dcSAM (Li et al. 2025) as shown in ASA dependent route in Fig. 2. Although early studies such as Tait (1976) suggested the existence of non-canonical polyamine-biosynthetic routes, the ASA-dependent pathway was biochemically elucidated only recently. Lee et al. (2009) identified carboxyspermidine dehydrogenase (CASDH) and carboxyspermidine decarboxylase (CASDC) in *Vibrio cholerae*, thereby establishing the modern understanding of the ASA-dependent spermidine biosynthetic pathway.　It is now known to be widespread among marine and host-associated Gram-negative bacteria, including species of *Campylobacter*, *Agrobacterium*, *Shewanella*, *Bacteroides*, and several Rhizobiales (Hanfrey et al. 2011; Kim et al. 2016; Li et al. 2025). In these organisms, the CASDH–CASDC pathway represents an alternative route for spermidine synthesis. More recently, ASA-dependent pathways have also been identified for synthesizing spermine and thermospermine. In these cases, spermidine is converted to carboxyspermine or carboxythermospermine, followed by decarboxylation to produce spermine or thermospermine, respectively (Lee et al. 2009; Hanfrey et al. 2011; Kim et al. 2016). These enzymes are not homologous to classical dcSAM-dependent spermine synthases, exemplifying convergent evolution in polyamine biosynthesis. Importantly, these alternative pathways can coexist with dcSAM-dependent reactions, generating hybrid polyamine biosynthetic networks. For example, an organism may use an ASA-dependent module upstream (Arg → Agm → *N*¹-aminopropylagmatine → Spd) by carboxyaminopropylagmatine dehydrogenase (CAPADH) and carboxyaminopropylagmatine decarboxylase (CAPADC) while using the classical dcSAM-dependent module downstream to synthesize spermine. This modularity creates flexible and diverse polyamine biosynthetic capacities (Fig. 2).

### Unique polyamine biosynthetic pathways in hyperthermophiles

As mentioned above, in both bacterial and archaeal domains, hyperthermophiles utilize a distinctive polyamine biosynthetic pathway that proceeds as follows: Arg → Agm→ *N*¹-aminopropylagmatine→ spermidine. In this pathway, aminopropylation precedes deimination, a reaction order that is reversed relative to the classical polyamine biosynthetic pathway. This modified pathway architecture represents a remarkable thermophile-specific evolutionary innovation.

In *Thermococcus kodakarensis*, *N*⁴-bis(aminopropyl)spermidine [3(3)(3)4] is the major intracellular polyamine and plays a critical role in supporting growth at temperatures approaching 90 °C. The biosynthesis of this branched-chain polyamine (BCPA) begins with spermidine as the initial substrate (Okada et al. 2014). The key enzyme in this process is BpsA (TK1691/*bpsA*), a dcSAM-dependent aminopropyltransferase that catalyzes two sequential aminopropylation reactions: first converting spermidine into *N*⁴-aminopropylspermidine, and subsequently converting this intermediate into *N*⁴-bis(aminopropyl)spermidine, as illustrated in the BCPA pathway shown in Fig. 2. Thus, whereas the canonical SpeE enzyme catalyzes a single aminopropyl transfer, BpsA performs two successive transfers using spermidine as the starting substrate.

Not all hyperthermophiles synthesize branched-chain polyamines. Hyperthermophilic archaea that lack BCPAs are predominantly found within the Crenarchaeota, including members of the genera *Pyrobaculum*, *Sulfolobus*, *Acidianus*, and *Metallosphaera*, in which linear polyamines such as norspermine and norspermidine constitute the major intracellular species (Hamana et al. 2022). *Pyrobaculum calidifontis* strain VA1, isolated from terrestrial hot springs (Amo et al. 2002), lacks BCPAs and instead produces the linear polyamine norspermine [333] (Fukuda et al. 2022). *P. calidifontis* possesses an aminopropyltransferase ortholog (*Pc*-SpeE) that preferentially supports norspermidine and norspermine production. *Pc*-SpeE exhibits high catalytic activity toward aminopropylagmatine and norspermidine, but shows low affinity for putrescine, which is not stably accommodated within its active site. In *P. calidifontis*, norspermidine [33] is produced predominantly via oxidative degradation of thermospermine [334] (Fukuda et al. 2022). The identity of the polyamine oxidase/dehydrogenase responsible for this conversion, however, remains unknown.

## Discovery of BpsA and its catalytic mechanism and structural insights

The search for the enzyme responsible for BCPA biosynthesis culminated in the identification of a novel aminopropyltransferase, BpsA (branched-chain polyamine synthase, EC 2.5.1.128). Okada et al. (2014) demonstrated that BpsA catalyzes the aminopropylation of spermidine to generate *N*⁴-aminopropylspermidine [3(3)4], which is further extended to the predominant BCPA, *N*⁴-bis(aminopropyl)spermidine [3(3)(3)4]. Biochemical and structural studies subsequently revealed that, unlike classical spermidine/spermine synthases that employ an SN2 displacement reaction (Wu et al. 2007, 2008), BpsA operates through a ping–pong Bi–Bi mechanism involving covalent aminopropyl–enzyme intermediates (Hidese et al. 2017b, 2019).

### Comparison with canonical aminopropyltransferases

Classical aminopropyltransferases such as spermidine synthase and spermine/thermospermine synthases catalyze the dcSAM-dependent transfer of an aminopropyl group to linear polyamines via a ternary-complex SN2 mechanism. In these systems, both dcSAM and the polyamine substrate are bound simultaneously, enabling direct nucleophilic attack by the deprotonated amino group on the Cα of the dcSAM aminopropyl moiety. No covalent enzyme–intermediate is formed in this process. In contrast, BpsA differs from canonical enzymes in two fundamental aspects: (i) it uses a ping–pong Bi–Bi mechanism instead of a ternary-complex SN2 mechanism, and (ii) its active-site architecture is larger, more flexible, and tailored to accommodate long and branched polyamines. Rather than transferring the aminopropyl group directly from dcSAM to the polyamine, BpsA first stores the aminopropyl group on acidic residues in the enzyme, forming transient ester-linked intermediates. Subsequently, the acceptor polyamine binds and receives the aminopropyl group in the second half-reaction. These features enable BpsA to process bulkier substrates and to introduce branched modifications in a stepwise manner, distinguishing it mechanistically and structurally from classical aminopropyltransferases.

### Catalytic features of branched-chain polyamine synthases

**Fig. 3:**
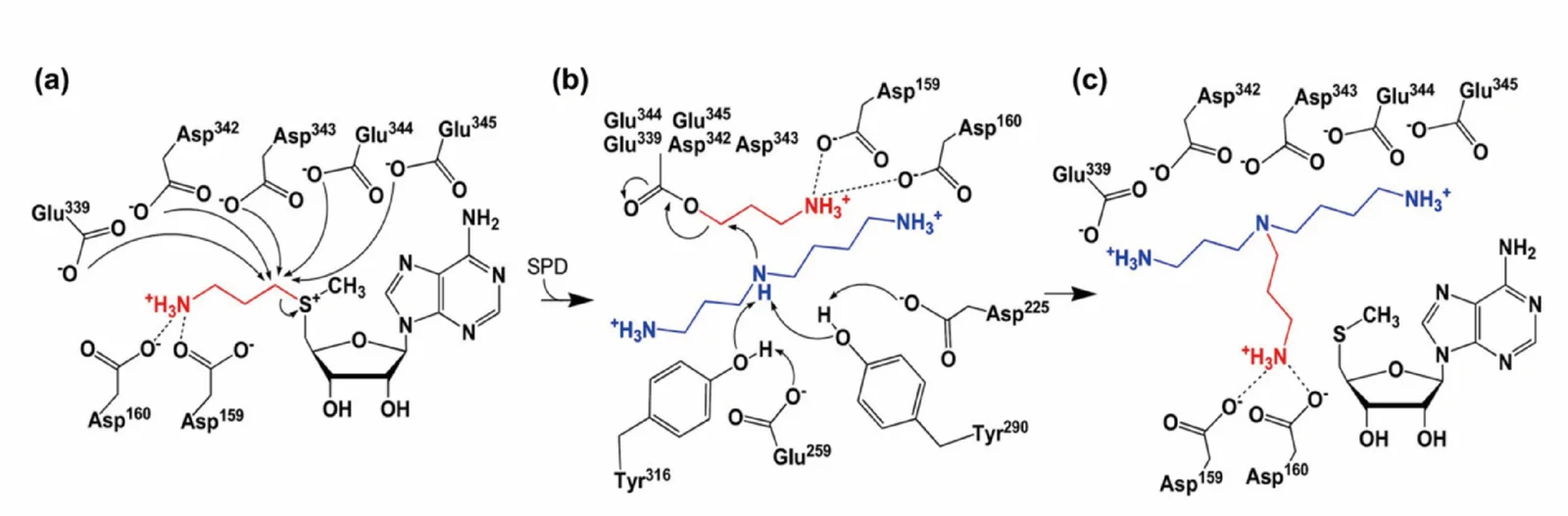
Catalytic mechanism of BCPA synthesis of *Tk*-BpsA. Proposed reaction mechanism of BpsA. **a** One of the five acidic residues (Glu339, Asp342, Asp343, Glu344, and Glu345) attacks the C-α atom of the dcSAM aminopropyl group. Asp159 and Asp160 are positioned to form a salt bridge with the aminopropyl moiety of dcSAM. **b** The amino group of spermidine, whose nucleophilicity is enhanced by neighboring acidic and aromatic residues (Asp225, Glu259, Tyr290, and Tyr316), then attacks the C-α atom of the aminopropyl group anchored to one of the acidic residues. **c** This reaction yields *N*⁴-aminopropylspermidine

Kinetic analyses of archaeal and bacterial BpsA homologs show parallel Lineweaver–Burk plots, consistent with a ping–pong Bi–Bi mechanism in which dcSAM and the polyamine substrate bind sequentially. In the first half-reaction, dcSAM reacts with the enzyme to generate MTA and an aminopropylated enzyme intermediate, while in the second half-reaction, the polyamine substrate receives the aminopropyl group. Structural, mutational, and LC–MS/MS analyses have revealed that BpsA uses conserved acidic residues as aminopropyl carriers. In *T. kodakarensis* BpsA (*Tk*-BpsA), LC–MS/MS demonstrated that multiple Glu/Asp residues within the flexible C-terminal acidic cluster function as the major aminopropyl–carrying sites. Earlier hypotheses proposed that Asp159 in the conserved GDDD motif served as the aminopropyl carrier. However, subsequent LC–MS/MS analysis showed that Asp159 instead plays a critical role in orienting and stabilizing the dcSAM aminopropyl moiety, while the actual carriers are the acidic C-terminal residues as shown in Fig. 3. Thus, BpsA catalysis proceeds via a two-step relay reaction—dcSAM → enzyme → polyamine—rather than the single-step SN2 displacement typical of classical polyamine synthases. (Hidese et al. 2017b)

A second hallmark of BpsA catalysis is its enlarged, negatively charged active-site cavity combined with a flexible C-terminal lid-like region. This region transiently captures aminopropyl groups and presents them to the *N*⁴ position of spermidine-derived substrates. C-terminal swap experiments between *T. kodakarensis* and *T. thermophilus* BpsA confirmed that this region governs substrate selectivity and the ratio of BCPA products, underscoring its role as a modular determinant of product specificity (Hidese et al. 2019). Details of these two BpsAs are mentioned as below.

### *Tk-*BpsA (hyperthermophilic archaeal type) and *Tt-*BpsA (thermophilic bacterial type)

#### *Tk*-BpsA from *Thermococcus kodakarensis*: a “specialist-type” BpsA

The BpsA enzyme from *T. kodakarensis* represents a “specialist-type” aminopropyltransferase optimized for producing *N*⁴-bis(aminopropyl)spermidine [3(3)(3)4]. *Tk*-BpsA catalyzes two sequential reactions:

1. spermidine → *N*⁴-aminopropylspermidine,
2. *N*⁴-aminopropylspermidine → *N*⁴-bis(aminopropyl)spermidine.

Kinetic analyses indicate that the first reaction step is rate-limiting, whereas the second proceeds rapidly once the intermediate is formed. *Tk-*BpsA displays strong preference for longer linear polyamines, including spermidine, spermine, and *N*⁴-aminopropylspermidine, and comparatively weak activity toward norspermidine and short-chain diamines. The conserved GDDD motif (Gly157–Asp158–Asp159–Asp160 in *Tk-*BpsA) is indispensable for catalysis. Mutational studies confirmed the essential roles of Asp159 and Asp160 in positioning the dcSAM aminopropyl group. LC–MS/MS mapping identified numerous C-terminal acidic residues carrying aminopropyl esters, confirming that the C-terminal acidic cluster represents the true aminopropyl carrier region. Physiologically, the essentiality of *Tk*-BpsA is underscored by the fact that *ΔbpsA* mutants fail to grow at 93 °C, demonstrating that *Tk-*BpsA-dependent 3(3)(3)4 is critical for hyperthermophilic survival. For these reasons, *Tk*-BpsA is considered a “specialist-type” BpsA, characterized by a narrow substrate preference and a dominant product profile focused on a single major BCPA.

#### *Tt*-BpsA from *Thermus thermophilus*: a “generalist-type” BpsA

In contrast, the BpsA from *T. thermophilus* functions as a “generalist-type” enzyme capable of generating a broader mixture of BCPAs. *Tt-*BpsA efficiently synthesizes both 3(3)(3)4 and 3(3)(3)3, and displays high activity toward spermidine-, spermine-, and norspermidine-derived substrates. As a result, *T. thermophilus* maintains a more diverse intracellular BCPA profile, which may contribute to its adaptation to aerobic thermophilic environments. Although LC–MS/MS mapping of aminopropylated residues has not yet been conducted for *Tt-*BpsA, the presence of a conserved DEE-containing acidic motif in the C-terminal region, together with structural similarities to *Tk-*BpsA, strongly suggests that *Tt-*BpsA uses analogous acidic C-terminal residues as aminopropyl carriers. C-terminal swap experiments between archaeal and bacterial BpsA homologs demonstrate that this short flexible region is a tunable determinant of substrate preference and product distribution (Hidese et al. 2019), supporting its modular evolutionary role.

Accordingly, *Tt-*BpsA is referred to as a “generalist-type” BpsA, defined by its broader substrate spectrum and production of multiple BCPA species, in contrast to the specialization observed in *Tk*-BpsA.

## Physiological functions of BCPAs

### BCPAs as thermophile-specific polyamines

In microorganisms, polyamines enhance DNA and RNA stability, support ribosome function, and promote resistance to diverse stresses (Tabor and Tabor 1985; Wallace et al. 2003). In hyperthermophiles, branched-chain polyamines (BCPAs), synthesized by the dcSAM-dependent aminopropyltransferase BpsA (TK1691/bpsA), play specialized roles in stabilizing macromolecular systems under extreme thermal conditions. Comparative genomic analyses further reveal that *bpsA* orthologs are present in both archaea and bacteria, yet their distribution is strikingly restricted to thermophilic and hyperthermophilic lineages. Together with the widely supported view that life originated in high-temperature environments, this pattern suggests that BpsA may represent a primitive form within the evolutionary history of aminopropyl group–transfer enzymes—an ancient innovation that emerged early and was selectively retained in organisms that continued to inhabit extreme thermal settings. No biosynthetic pathways capable of generating BCPAs are known in higher organisms. Taken together with the phylogenetic confinement of BpsA to thermophiles, these findings reinforce the view that BCPAs are highly specialized metabolites uniquely adapted to hyperthermophilic life.

Despite the apparent specialization of BCPAs for hyperthermophilic life, it is important to note that not all hyperthermophiles rely on branched-chain polyamines as mentioned in Sect. "A thermophile-specific pathway: agmatine → N¹-aminopropylagmatine → spermidine". Hyperthermophilic archaea that lack BCPAs are predominantly found within the Crenarchaeota (Hamana et al. 2022). Synthesis pathway is shown as norspermine (NSPM) route in Fig. 2. The physiological significance of these linear polyamines in supporting growth at extreme temperatures remains incompletely understood. However, insights from *Thermococcus kodakarensis* provide a useful framework for interpretation. A BCPA-deficient mutant of this organism can still grow at high temperatures, but its maximum growth temperature is reduced (Okada et al. 2014; Fujiwara et al. 2025a, 2025b), and it exhibits decreased tolerance to thermal fluctuation and other stresses compared with the wild type (Yamori et al. 2020). Notably, under these conditions the mutant shows a marked intracellular accumulation of spermidine, suggesting that elevated levels of linear polyamines can partially compensate for the absence of BCPAs, albeit not to the extent achieved by the branched-chain species. By analogy, hyperthermophiles that naturally lack BCPA biosynthetic capacity may achieve thermoprotection by maintaining high intracellular levels of linear polyamines such as norspermine or norspermidine, thereby functionally substituting—at least in part—for the higher efficiency of BCPAs at lower concentrations.　This comparison raises the possibility that hyperthermophiles employ at least two distinct but functionally convergent polyamine strategies to cope with extreme thermal stress: one based on low-abundance, high-efficiency branched-chain polyamines, and another relying on the abundant accumulation of linear polyamines. Such diversification in polyamine utilization may reflect alternative evolutionary solutions to the shared challenge of macromolecular stabilization at near-boiling temperatures.

### DNA binding and structural modulation

Because of their high charge density, BCPAs bind DNA more strongly than linear polyamines and protect nucleic acids from thermal denaturation. Muramatsu et al. (2016) showed that BCPAs induce cross-linked DNA meshworks and promote temperature-dependent B→A/C conformational transitions, while Nishio et al. (2018) demonstrated local DNA opening events not observed with spermidine or long-chain polyamines. These findings suggest that BCPAs both compact DNA and modulate DNA accessibility dynamically, potentially influencing transcription and replication under high-temperature conditions.

An acetylated form of BCPA was identified in *T. kodakarensis* (Hidese et al. 2017a). By analogy with histone acetylation, which reduces positive charge and promotes euchromatin formation, acetyl-BCPA may locally decrease DNA condensation, while unmodified BCPA may promote compaction. Although methyl modifications (such as H3K4me3) also contribute to euchromatin in eukaryotes, acetylation remains the most representative mark of open chromatin. Thus, post-synthetic modification of BCPA may represent a thermophilic strategy for regulating DNA accessibility, contributing to transcriptional control at high temperatures.

Consistent with these observations, Higashibata et al. (2000) demonstrated that spermine stabilized nucleosome-like structures formed with archaeal histones at elevated temperatures. Although the study did not specifically examine BCPAs, these results strongly suggest that BCPA, with even higher DNA-binding affinity and structural-modulating capacity than linear polyamines, likely contributes in vivo to the stabilization of chromatin-like architecture in *Thermococcus* and related hyperthermophiles. Such structural reinforcement would be expected to support both genome integrity and the proper regulation of transcription under extreme thermal conditions.

### Thermoadaptation and survival under fluctuating conditions

*T. kodakarensis*, originally isolated from coastal hot springs on Kodakarajima Island, experiences substantial natural temperature fluctuations. These hot springs form tide pools that are submerged at high tide and exposed at low tide. During winter, ambient temperatures can fall to almost 0 °C. Hyperthermophilic genera such as *Thermotoga* sp, which also produce BCPAs, inhabit the same environments. Under laboratory simulations of repeated 85 °C ↔ 0 °C cycles, the wild type maintains viability, whereas *bpsA* deletion mutant DBP1 loses viability after two cycles and forms large cellular aggregates (Fujiwara et al. 2025a). DBP1 can grow at 85 °C, but growth is severely impaired at 93 °C, demonstrating that BCPA is indispensable near the upper thermal limit. Although spermidine accumulates at high levels in DBP1, it cannot compensate for the absence of BCPA (Yamori ei al. 2020; Okada et al. 2014). DBP1 is also hypersensitive to the thermostable biosurfactant sophorolipid, suggesting impaired membrane stability. Together, these observations indicate that BCPA is crucial not only for high-temperature survival but also for maintaining membrane integrity, cellular morphology, and resilience to hot–cold cycling.

### Transcription and translation

#### Stabilization of RNA polymerase and ribosomes

Biochemical analyses revealed that BCPA stabilizes archaeal RNA polymerase (RNAP) and enhances its transcriptional activity at high temperatures (Yamori et al. 2020). BCPA is effective at concentrations an order of magnitude lower than spermidine and dramatically protects RNAP from thermal inactivation at 90 °C. Co-purification of multiple ribosomal proteins with RNAP suggests that BCPA promotes tight coupling between transcription and translation, which is essential for efficient gene expression in hyperthermophiles. In *T. thermophilus*, LCPA and BCPA are required for ribosome stability and maintenance of specific tRNA species (Nakashima et al. 2017). Consistent with these findings, *bpsA* deletion mutant DBP1 shows reduced levels of several tRNAs (Fukuda et al. 2020), implying that BCPA contributes to translation fidelity and robustness by stabilizing components of the translational apparatus.

#### Gene-specific regulation

Genome-wide omics analyses revealed extensive BCPA-dependent regulation. RNA-seq at 90 °C showed that 424 genes are upregulated and 21 are downregulated in *bpsA* deletion mutant DBP1 relative to the parental strain KU216 (Fukuda et al. 2020). Many upregulated genes encode metabolic enzymes, including 2-oxoacid: ferredoxin oxidoreductase, suggesting broad metabolic remodeling in the absence of BCPA. A striking observation is the discrepancy between mRNA and protein levels for several genes. Flagellin proteins FlaB2–4 and the hydrogenase subunit HyhL are undetectable in DBP1 despite equal or elevated transcript levels. Electron microscopy confirmed the loss of flagella, and Western blotting confirmed the absence of HyhL (Fukuda et al. 2020). These results indicate that BCPA affects gene expression at multiple levels—transcriptional, post-transcriptional, translational, and post-translational. Notably, the transcript of TK0134—a predicted histone deacetylase ortholog—is essentially undetectable in the strain DBP1, indicating that its expression is strongly BCPA-dependent. Given that acetylated BCPA has been identified in *T. kodakarensis* (Hidese et al. 2017a), TK0134 may function in regulating the cellular level of acetyl-BCPA, although this remains speculative. Elucidating whether TK0134 directly modulates BCPA acetylation–deacetylation dynamics represents an intriguing direction for future study.

### Membrane stabilization

BCPAs are abundant not only in the cytosol but also in the membrane fraction of *T. kodakarensis*, particularly during stationary phase. DBP1 exhibits hypersensitivity to detergents and biosurfactants, and rapidly loses viability during hot–cold cycling (Fujiwara et al. 2025a). These observations parallel early studies showing that polyamines stabilize lipid bilayers (Schuber 1989). Thus, BCPAs likely interact with anionic archaeal lipids and stabilize membrane-associated protein complexes, contributing directly to membrane robustness.

### Functional replacement of BCPA by thermospermine

To investigate whether other polyamines can substitute for BCPA, *bpsA* in *T. kodakarensis* of parental KU216 (*ΔpyrF*) was replaced with *speE* from the hyperthermophilic crenarchaeon *Pyrobaculum calidifontis*, yielding strain KPS (*ΔbpsA*::*Pc-speE ΔpyrF*) (Fukuda et al. 2022; Fujiwara et al. 2025a). *Pc*-SpeE synthesizes norspermidine [33] and norspermine [333] in vivo, and produces thermospermine [334] in vitro from spermidine and dcSAM (Fukuda et al. 2022). In *T. kodakarensis*, *Pc-*SpeE predominantly generates thermospermine, which becomes the major polyamine in stationary phase, whereas spermidine levels remain unchanged (Fujiwara et al. 2025a). Physiologically, thermospermine partially restores BCPA functions: (1) At 85 °C, KPS shows wild-type–like growth. (2) At 93 °C, KPS grows more slowly than KU216 but clearly better than DBP1. (3) Under repeated hot–cold cycling, KPS shows intermediate survival, better than DBP1 but below parental KU216. (4) KPS shows near wild-type biosurfactant (sophorolipid) tolerance, indicating restoration of membrane robustness.

At the molecular level, *hyhL* expression is restored in KPS across a broad temperature range, and in vitro translation with cell-free extracts shows that thermospermine stimulates HyhL synthesis but at sub-millimolar concentrations, over a wider effective range than BCPA. Spermidine requires much higher concentrations to produce similar effects. These findings imply that thermospermine can mimic key functional aspects of BCPA, perhaps by adopting similar bent conformations or binding modes with nucleic acids and translation machinery.

### Integrated view

Collectively, these studies demonstrate that BCPA is a central determinant of stress resilience in hyperthermophiles, contributing to: (1) stabilization of DNA, RNA, RNAP, ribosomes, and membranes, (2) modulation of chromatin-like structures, (3) maintenance of transcription–translation coupling, (4) regulation of gene expression at multiple levels, (5) preservation of cell integrity during extreme thermal fluctuations. Thermospermine can compensate for many—but not all—BCPA functions. From an evolutionary perspective, BpsA-dependent BCPA biosynthesis represents a thermoadaptive extension of the classical polyamine pathway, integrated into the regulatory and stress-response networks of hyperthermophiles.

## Applications and prospects

### Overview

Polyamines have long been exploited as versatile cationic tools for DNA condensation, membrane interaction, and nucleic acid stabilization. BCPAs, owing to their distinctive branched structures and enhanced nucleic-acid-binding properties, may extend these applications, although limited availability and high synthesis cost currently constrain large-scale use. Their most promising utility lies in niche, high-value applications where performance outweighs cost considerations.

### Gene delivery

Lipopolyamines and polyethylenimine (PEI)-based polymers are well-established gene delivery agents (Remy et al. 1998; Byk et al. 1998; Ewe et al. 2014). Spermine has also been shown to increase the transfection efficiency of cationic polymeric gene vectors (Lv et al. 2025). Incorporating branched moieties such as BCPAs could further enhance DNA binding, condensation capacity, and the stability of polyplexes. However, excessively tight binding may impede intracellular DNA unpacking and release, a major limitation of many cationic polymer–based transfection systems. Thus, successful application of BCPAs in gene delivery will likely require controlled-release or stimuli-responsive designs that balance strong DNA protection with efficient cytoplasmic availability of the genetic payload.

### Nucleic acid capture, concentration, and detection

**Fig. 4:**
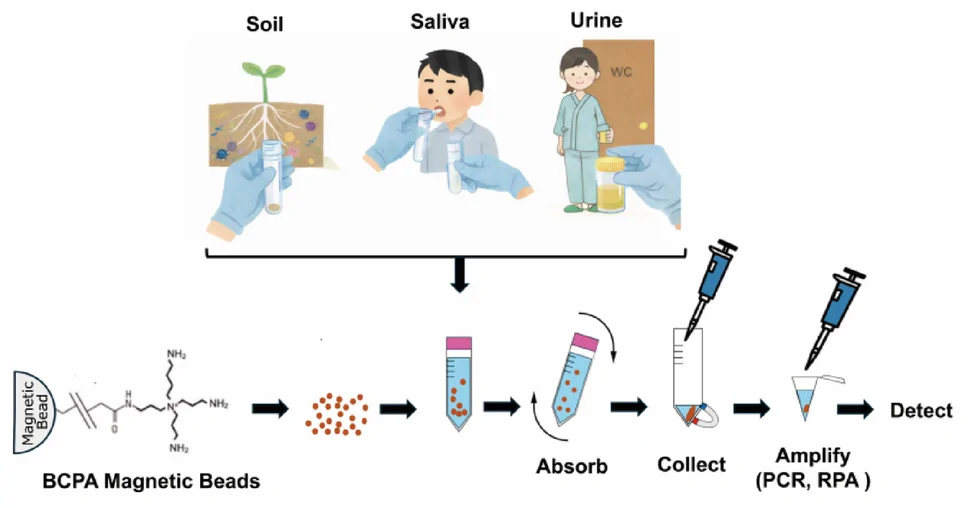
Schematic overview of DNA capture, release, and amplification using BCPA-conjugated magnetic beads. DNA from environmental and biological samples (soil, oral, and urine specimens) is captured by BCPA beads. The addition of dNTPs and pyrophosphate promotes dissociation of DNA from the beads. Pyrophosphate generated during amplification may further assist DNA release and mitigate magnesium chelation, thereby improving amplification efficiency

PEI, silica matrices, and chaotropic-salt–dependent columns are widely used for nucleic acid purification, but their performance often declines in dilute, inhibitor-rich, or high-volume samples. Because BCPAs show exceptionally high affinity for DNA, their immobilization on solid supports offers a powerful alternative. The only published application to date is the use of BCPA-conjugated magnetic beads, which enabled the efficient recovery and direct PCR detection of trace DNA from aqueous solutions (Fujiwara et al. 2025b). The combination of recombinase polymerase amplification (RPA), a type of isothermal amplification, with BCPA beads enabled the reliable detection of approximately 10² target molecules from a volume of 10 mL of saline (Nishi et al. 2026). In these proof-of-concept experiments, BCPA beads outperformed spermidine- and silica-based matrices, achieving higher recovery from dilute solutions.　Although synthesis cost prevents large-scale adoption, BCPA-based capture systems hold strong potential for specialized applications, including: (1) environmental DNA (eDNA) surveys, (2) forensic or trace-level DNA recovery, (3) pathogen detection in complex biological fluids, (4) low-copy nucleic acid extraction prior to PCR and isothermal amplification. Dissociation of DNA from BCPA-conjugated magnetic beads exhibited unique characteristics. Neither SDS nor sodium chloride effectively dissociated DNA from the beads, suggesting extremely strong electrostatic interactions. Remarkably, DNA was efficiently released upon addition of nucleotides and pyrophosphate. Leveraging these properties, BCPA beads containing recovered DNA were directly introduced into PCR reaction mixtures. During PCR, pyrophosphate generated as a byproduct may further facilitate DNA release and can inhibit DNA synthesis by chelating magnesium ions essential for polymerase activity. However, it is likely sequestered by BCPA beads, preventing magnesium depletion and potentially improving PCR efficiency (Fig. 4).

### Antimicrobial and antibiofilm potential of polyamines and polyamine-derived materials

Polyamines interact dynamically with nucleic acids, membranes, and extracellular polymeric substances; accordingly, many studies have examined whether these physicochemical properties translate into antibacterial or antibiofilm activity. Natural polyamines such as spermidine and spermine generally exhibit limited direct antimicrobial effects. However, norspermidine has been reported to inhibit or disperse biofilms in several organisms, including *Staphylococcus aureus* and *Pseudomonas aeruginosa* (Qu et al. 2016; Cardile et al. 2017). These effects appear to involve disruption of extracellular polymer matrices and alterations in surface-associated gene expression.　Nonetheless, proposed norspermidine-mediated biofilm disassembly in *Bacillus subtilis* was later refuted (Hobley et al. 2014), underscoring the species- and context-dependent nature of polyamine-associated phenotypes. Exogenous norspermidine and spermidine inhibit biofilm formation in *Escherichia coli* but not in *Salmonella enterica*, and neither polyamine promotes biofilm disassembly (Nesse et al. 2015). Importantly, these authors emphasized that polyamine-induced alkalization of the culture medium can strongly influence observed outcomes, highlighting the need to control pH to avoid misinterpretation.

In contrast to natural polyamines, stronger and more reproducible antimicrobial activities have been observed for synthetic polyamine analogs, particularly those engineered to increase cationic density or incorporate hydrophobic substituents. Rationally designed linear synthetic polyamines display potent bactericidal activity through rapid membrane depolarization and permeabilization (Douglas et al. 2022). Polyamine–bioactive conjugates further extend this concept, inhibiting both Gram-positive and Gram-negative bacteria by enhancing membrane disruption relative to their parent molecules (Inclán et al. 2023). Cationic polymers inspired by polyamine chemistry represent another major class of antimicrobial materials. Polytrimethylenimine (PTMI) exhibits strong antibacterial activity through multivalent interactions with the bacterial envelope, leading to membrane rupture (Pachla et al. 2023). Polyethylenimine (PEI), when immobilized on surfaces, forms durable contact-active antimicrobial coatings, enabled by its high charge density and flexible branched architecture (Gibney et al. 2012; Hernandez-Montelongo et al. 2017). Polyamidoamine dendrimers also provide highly charged multivalent scaffolds with antibacterial and antibiofilm properties (Calabretta et al. 2017; Śmigiel-Gac et al. 2024).

An emerging category of interest is polyamine-derived nanomaterials. Carbon quantum dots synthesized from biogenic polyamines (CQD–PAs) exhibit broad-spectrum antibacterial activity against both Gram-positive and Gram-negative organisms (Jian et al. 2017). Their highly charged nanosurfaces facilitate strong electrostatic binding to bacterial membranes and subsequent membrane disruption.

Although no peer-reviewed studies have yet evaluated authentic BCPAs for antimicrobial or antibiofilm activity, their physicochemical properties strongly suggest such potential. BCPAs possess higher cationic density, stronger DNA and membrane binding, and more rigid three-dimensional geometries than linear polyamines. These features are precisely those that underpin the antibacterial performance of synthetic polyamine analogs, cationic polymers, and polyamine-derived nanomaterials. Consequently, BCPAs - either in free form or immobilized on solid supports such as magnetic or polymeric beads - may likewise disrupt bacterial membranes, interfere with extracellular polymer matrices, or prevent biofilm maturation. Systematic evaluation of BCPAs in these contexts therefore represents an important direction for future research.

## Conclusion

Branched-chain polyamines (BCPAs) represent a unique class of polyamines restricted primarily to thermophiles, although they are not universally distributed across all hyperthermophilic organisms and are not exclusive to archaea. Their occurrence depends strictly on the presence of the *bpsA* gene, which encodes the novel dcSAM-dependent aminopropyltransferase responsible for generating branched polyamine structures. Functional studies in *Thermococcus kodakarensis* have demonstrated that Δ*bpsA* mutants lose tolerance to fluctuating temperature stress, exhibit broad defects in gene expression, and display strongly compromised membrane robustness. Replacement of *bpsA* with the *speE* gene from *Pyrobaculum calidifontis* partially restores growth and stress resistance through the intracellular production of thermospermine, revealing that alternative long-chain polyamines can compensate for several, but not all, physiological functions of BCPAs. These findings, together with the presence of norspermine and thermospermine in *Pyrobaculum*, suggest that BCPAs, thermospermine, and norspermine form a functionally overlapping triad of long-chain polyamines essential for the survival of diverse thermophiles. Their roles extend far beyond simple nucleic acid stabilization. Rather, they involve size- and charge-dependent interactions with archaeal membranes, dynamic modulation of DNA and RNA architecture, stabilization of ribonucleoprotein complexes, and regulation of transcription–translation coupling. BCPAs therefore serve as central integrators of cellular stress physiology, enabling hyperthermophiles to withstand high temperatures and extreme thermal fluctuations.

Beyond their physiological significance, BCPAs have only recently been applied to biotechnology. Immobilization of BCPAs onto magnetic beads enables the ultrasensitive recovery and detection of low-copy DNA from aqueous solutions, outperforming conventional spermidine- and silica-based systems. Although the high synthesis cost of BCPAs currently limits broad application, their distinctive physicochemical properties point to a future in niche, high-value contexts such as environmental DNA analysis, forensic diagnostics, and novel nucleic acid concentration or isothermal amplification workflows. In addition, their branched, compact, and highly cationic nature suggests potential relevance to antimicrobial surfaces or polyamine-inspired biomaterials, though further evaluation is required.

In conclusion, BCPAs occupy a distinctive and multifaceted position among polyamines. They illuminate fundamental principles of thermophile adaptation, exemplify a novel branch of aminopropyltransferase enzymology through BpsA, and present promising opportunities for specialized biotechnological applications. Continued investigation of their biosynthesis, structural biology, and functional diversity will be essential to fully unlock the potential of these extraordinary molecules.

No peer-reviewed studies have evaluated authentic branched-chain polyamines (BCPAs) themselves for antimicrobial activity. However, given their strong cationic nature, compact topology, and DNA-binding capacity, BCPAs may share physicochemical characteristics with potent antimicrobial polyamine-based materials. Future investigations are needed to determine whether BCPAs have intrinsic antimicrobial or antibiofilm properties, either alone or when immobilized on solid supports.

## Data Availability

No datasets were generated or analysed during the current study.

## Abbreviations

Agm: Agmatine
AdoMetDC: S-adenosylmethionine decarboxylase
AIH: Agmatine iminohydrolase (agmatine amidinohydrolase) (EC 3.5.3.12)
ASA: Aspartate β-semialdehyde
BCPA: Branched-chain polyamine
BpsA: Branched-chain polyamine synthase
CAPADC: Carboxyaminopropylagmatine decarboxylase
CAPADH: Carboxyaminopropylagmatine dehydrogenase
CASDC: Carboxyspermidine decarboxylase
CASDH: Carboxyspermidine dehydrogenase
dcSAM: Decarboxylated S-adenosylmethionine
DBP1: ΔbpsA mutant strain of *Thermococcus kodakarensis*
dNTP: Deoxyribonucleoside triphosphate
eDNA: Environmental DNA
FlaB2–4: Flagellin proteins
GC–MS: Gas chromatography–mass spectrometry
HPLC: High-performance liquid chromatography
HPGC–MS: High-performance gas chromatography–mass spectrometry
HyhL: Large subunit of membrane-bound [NiFe]-hydrogenase
KPS: *ΔbpsA*::*Pc-speE* strain of *Thermococcus kodakarensis*
KU216: Standard laboratory strain of *Thermococcus kodakarensis*
LC–MS/MS: Liquid chromatography–tandem mass spectrometry
LCPA: Long-chain polyamine
MTA: 5′-methylthioadenosine
NSPD: Norspermidine
NSPM: Norspermine
N*-*CPA: *N*-carbamoylputrescine amidohydrolase (EC3.5.1.53)
ODC: Ornithine decarboxylase
OR: 2-oxoacid:ferredoxin oxidoreductase
*Pc*-SpeE: Spermidine synthase homolog from *Pyrobaculum calidifontis*
PCR: Polymerase chain reaction
PdaD: Arginine decarboxylase (TK0149)
PEI: Polyethylenimine
RPA: Recombinase polymerase amplification
RNAP: RNA polymerase
SpeA: Arginine decarboxylase
SpeB-like: Agmatine ureohydrolase–like enzyme (TK0882)
SpeC: Ornithine decarboxylase
SpeD: S-adenosylmethionine decarboxylase
SpeE: Spermidine synthase/aminopropyltransferase (TK0147)
SPM: Spermine
SPD: Spermidine
*T. kodakarensis*: *Thermococcus kodakarensis*
*T. thermophilus*: *Thermus thermophilus*
Tspm: Thermospermine
TK0134: Histone deacetylase–like protein
*Tt-*BpsA: BpsA from *Thermus thermophilus*
*Tk-*BpsA: BpsA from *Thermococcus kodakarensis*
